# A bioelectronic route to compassion: Rationale and study protocol for combining transcutaneous vagus nerve stimulation (tVNS) with compassionate mental imagery

**DOI:** 10.1371/journal.pone.0282861

**Published:** 2023-03-13

**Authors:** Sunjeev K. Kamboj, Matthew Peniket, Louise Simeonov

**Affiliations:** Clinical Psychopharmacology Unit, Research Department of Clinical, Educational and Health Psychology, University College London, London, United Kingdom; Osaka Metropolitan University: Osaka Koritsu Daigaku, JAPAN

## Abstract

**Background:**

The vagus nerve (VN) is a neural nexus between the brain and body, enabling bidirectional regulation of mental functioning and peripheral physiology. Some limited correlational findings suggest an association between VN activation and a particular form of self-regulation: compassionate responding. Interventions that are geared towards strengthening *self*-compassion in particular, can serve as an antidote to toxic shame and self-criticism and improve psychological health.

**Objective:**

We describe a protocol for examining the role of VN activation on ‘state’ self-compassion, self-criticism, and related outcomes. By combining transcutaneous vagus nerve stimulation (tVNS) with a brief imagery-based self-compassion intervention, we aim to preliminarily test additivity versus synergy between these distinct bottom-up and top-down methods for putatively regulating vagal activity. We also test whether the effects of VN stimulation accumulate with daily stimulation and daily compassionate imagery practice.

**Methods:**

Using a randomized 2 x 2 factorial (stimulation x imagery condition) design, healthy volunteers (n = 120) receive active (tragus) or sham (earlobe) tVNS plus standardized (audio-recorded) self-compassionate or sham mental imagery instructions. These interventions are delivered in a university-based psychological laboratory in two sessions, one week apart, as well as being self-administered between sessions by participants at home. Pre-stimulation, peri-stimulation and post-imagery measures of state self-compassion, self-criticism and related self-report outcomes are assessed in two lab sessions, separated by a week (Days 1 and 8). Heart rate variability is used as a physiological metric of vagal activity and an eye-tracking task assesses attentional bias to compassionate faces during the two lab sessions. On Days 2–7, participants continue their randomly assigned stimulation and imagery tasks at home, and complete state measures at the end of each remote session.

**Discussion:**

Demonstrating modulation of compassionate responding using tVNS would support a causal link between VN activation and compassion. This would provide a basis for future studies of bioelectronic approaches to augmenting therapeutic contemplative techniques.

**Clinical trials registration:**

ClinicalTrials.gov, Identifier: NCT05441774 (Date: July 1^st^ 2022).

**OSF registration:**

https://osf.io/4t9ha.

## Introduction

The mammalian nervous system has evolved to support caring and nurturing behaviors. In primates, in particular, an underlying caregiving system orchestrates complex and prolonged nurturing behaviors towards offspring. This system has extended functions in humans, enabling care-giving to be expressed towards individuals outside the immediate kinship group [1]. The ‘extended caregiving system’ is also thought to underlie a sensitivity to the suffering of others, as well as the uniquely human capacity for, and motivation to, alleviate this suffering [2]. This is a defining feature of the complex affective-cognitive-motivational state (and disposition) that is referred to as ‘compassion’ [3]. In this article, we describe a protocol and rationale for modulating compassionate responding through the interaction between a biological intervention (transcutaneous vagus nerve stimulation; tVNS) and a behavioural manipulation (compassionate mental imagery).

### Compassion, self-compassion and psychopathology

In addition to being an innate capability, a tendency towards compassionate responding is a trainable attribute [4]. Descriptions of such training, and its benefits, are often attributed to (practitioners of) the Mahayana branch of Buddhism [5, 6]. This tradition has strongly influenced and deepened our contemporary understanding of compassion, and provided a basis for the development of compassion-focused meditation approaches, which have become increasingly mainstream and accessible, even to those with no previous background in these practices [7]. Compassionate behavior (including the subjective state of compassion) is typically understood as being directed outwards, toward other (suffering) beings. However, the value of self-compassion as a cognitive-emotion regulation strategy and an antidote to pathological self-criticism is also increasingly recognized [8]. Certain prevalent social practices (e.g. maladaptive upward social comparisons [9]) breed a kind of toxic self-criticism and shame, which in turn, are thought to contribute to the increasing prevalence of anxiety and depression, despite improved availability of treatments for these common mental health problems [10]. Established psychological treatments (e.g. ‘standard’ cognitive behavioral therapy), are generally ill-equipped to deal with this increasingly prevalent transdiagnostic phenomenon of toxic self-criticism. These limitations of extant psychological therapies have motivated the development of a ‘third wave’ of behavioural treatments which have tended to incorporate theoretical and technical insights from Buddhist psychology [11]. For example, interventions that specifically aim to foster self-compassion to counteract toxic shame and harmful self-criticism have been developed for a range of psychological disorders. Although this field is still in its infancy and the likely true effect size of compassion-focused therapies remains to be determined [12], early syntheses of the evidence suggest promising effects across a variety of psychiatric disorders and outcomes [13]. Nonetheless, as with all psychological treatments, strategies for augmenting compassion-focused therapies may be required to optimize their efficacy, or to improve outcomes for specific patient groups. Recently there has been growing interest in augmentation of psychological therapies using neurostimulation [14]. In the case of compassion-focused therapies, neurostimulation strategies that modulate the *vagus nerve* might be especially appropriate as adjunctive treatments with behavioural and cognitive psychotherapies.

### The vagus nerve and self-regulation

The vagus nerve (VN)—a major component of the parasympathetic nervous system, and the nexus between peripheral organs and the brain—is largely comprised of sensory afferents [15], feeding brainstem structures with information about bodily states [16]. However, the VN also contains efferents originating from nuclei in the medulla that regulate cardiac, pulmonary and gastrointestinal activity. Optimal regulation of this bidirectional central-peripheral traffic—which includes the interaction between brain-stem structures, elements of the central autonomic network and the prefrontal cortex—is thought to be associated with mental well-being and has a role in a variety of emotion-regulation functions [17]. These include basic cognitive-affective capabilities such as evaluating facial expressions of emotions [18], and, based on theoretical accounts, more complex social-regulatory functions, such as generating feelings of safety [19] and promoting social engagement [20]. We direct interested readers to authoritative reviews summarizing the proposed role of the VN in social-cognitive-affective functioning [20, 21].

The VN has a critical role in regulating breathing and modulating heart rate, momentary fluctuations in which, partly reflect this modulatory influence. In fact, ‘heart rate variability’ (HRV) is a widely recognized biomarker of VN functioning and, by extension, emotion regulation [22]. Because self-compassion is itself viewed as a form of emotion regulation [23], it is perhaps unsurprising that (linear) associations between compassionate responding and HRV have been reported in a number of studies (see [24]). Although most of these studies have reported a positive association between compassion and HRV [24], many had small sample sizes, lacked adequate control conditions, used measures with unknown reliability, and did not pre-specify the relationships of interest. As such, the overall robustness of these findings remains unclear. Indeed, related studies on a variety of positively valenced, low-arousal emotional states (e.g. safeness, contentment, warmth) and positive affective dispositions (e.g. agreeableness, gratitude, meaning in life) suggest a quadratic rather than linear association with HRV [25–30]. This may also be true for HRV’s association with compassion, although few studies have examined this possibility (c.f. [27]).

To date, experimental studies examining the relationship between vagal activation and positive affect—including compassion—have tended to use top-down cognitive-behavioral strategies to induce/enhance these states while measuring HRV(e.g. [31–33]). In addition, however, bottom-up methods for activating the VN have been proposed [17, 34]; these tend to involve some form of regulated breathing or HRV biofeedback. Although such methods have been shown to influence compassion-adjacent behaviors (i.e. altruism [35]), we are not aware of published studies that have specifically examined the effects of such bottom-up behavioral VN regulation techniques upon compassionate responding.

### Vagus nerve stimulation

In fact, until relatively recently, direct manipulation of VN activity was only achieved through invasive means (requiring surgical implantation of electrodes on the VN). However, the past two decades have witnessed rapid developments in non-invasive VN stimulation technologies, including transcutaneous stimulation of the VN (tVNS). This non-invasive approach is possible because of the superficial nature of vagal innervation of parts of the face and neck, including the ear. This allows mild current pulses to be safely and painlessly applied to the auricular branch of the VN through the skin of the tragus or concha and cymba conchae of the external ear. An important feature of vagal innervation of the external ear in particular is that these nerve fibers do not extend far beyond the specified areas [16]. Not only does this provide an opportunity for anatomically-targeted stimulation, but also allows stimulation of nearby areas (e.g. the earlobe) to serve as a credible sham condition (i.e. credible to participants receiving a tVNS ‘intervention’).

Given the proposed role of the VN in regulating heart rate (variability), HRV would appear to serve as a positive control and bioassay for effective (i.e. ‘successful’) vagal modulation. Additionally, through the locus coeruleus-noradrenergic pathway, tVNS might influence attentional mechanisms, the activation of which can be assessed using pupillometry [36] and other measures of attention and attentional bias (eye fixations and dwell times) in response to salient stimuli (e.g. positively valenced facial expressions). As such, various psychophysiological indices may provide objective and non-intrusive means for assessing the degree of vagal modulation by tVNS.

### The ‘compassionate vagus’?

Beyond these advances in the psychophysiology of compassion, recently developed measures that are based on consensus definitions of compassion, enable (self-)compassionate ‘traits’ [37], and ‘states’ [38] to be reliably assessed. In addition, stimuli that appear to tap general ‘compassion sensitivity’ and are less reliant on introspective reports have also recently been developed (e.g. facial expressions of compassion; [39]). These various experimental advances now provide an opportunity to address—at least preliminarily—questions related to the sufficiency of VN activity in mediating compassionate responding [20]. Such peripheral, VN-mediated (bottom-up) control over complex psychological capabilities may be possible because of the bidirectional influence of VN-mediated physiological states on brain activity [17]. Although there has been a considerable amount of theoretical discussion about the role of the VN in compassion/compassionate behavior [20, 21], the empirical evidence for such a role is currently very limited [24].

### Experimentally manipulating vagal activity to determine causality in compassionate responding: Study rationale

Taken together, the developments in autonomic and contemplative science, and non-invasive methods for stimulating the VN, provide a basis for testing the effects of tVNS on compassionate responding (and related outcomes). We therefore outline a study protocol for an experiment that is intended to preliminarily examine the separate and combined effects of tVNS and self-compassionate mental imagery on self-compassion, self-criticism and related outcomes (state mindfulness, positive affect and HRV). In some respects, this may appear to be a high-risk approach: the main variables of interest (self-compassion and self-criticism) are assessed using self-report measures rather than fine-grained computationally-derived metrics [40], or objective outcomes measured with millisecond precision [41], which might be more likely to detect weak signals. However, while small effects can have important mechanistic implications, their direct clinical or other applied relevance may be more limited. The adoption of bioelectronic stimulation methods—for example in clinical psychological settings—will rely on convincing demonstrations of efficacy on clinically relevant outcomes—such as those used here. Nonetheless, we will also assess ‘compassion-sensitivity’, using high-precision eye-tracking metrics, although these are secondary outcomes.

Importantly, in addition to examining the stand-alone effects of tVNS on self-compassion and related outcomes, we will test the effects of combining tVNS with a behavioral intervention (compassionate mental imagery) that itself reliably increases subjective self-compassion [33, 42–44]. In fact, according to a psychophysiological model of compassion [20], vagal activation generates the physiological conditions *necessary* for compassionate responding, but is *insufficient* for generating such responding. As such, participants who undergo active tVNS and a sham imagery intervention, rather than the active tVNS and self-compassion imagery intervention might be expected to show minimal effects on self-compassion. Alternatively, those who receive the combined tVNS + self-compassion intervention—in which tVNS is hypothesized to produce the appropriate physiological conditions for (self-)compassion—would show the largest responses (e.g. increased subjective self-compassion and decreased self-criticism) following stimulation.

By examining the effects of tVNS longitudinally, with repeated stimulation (active vs. sham) and mental imagery (active vs. sham) practice over the course of a week, we will also determine whether any augmenting effects on self-compassion and related outcomes are ‘dose-dependent’, becoming more evident after repeated stimulations. Cumulative effects might be expected to emerge if—in addition to providing an acute neurophysiological receptivity to compassion—tVNS also produces a central physiological state that is conducive to neuroplasticity [45]. If so, such effects would again only be expected if tVNS has an appropriate ‘behavioral substrate’ (i.e. the self-compassion intervention) upon which to act (i.e. the effects of stimulation are task- and use-dependent [45]). In the current study, the compassionate mental imagery intervention serves the role of this behavioural substrate. As a separate, confirmatory line of investigation, we will test the presence and nature (e.g. linear v quadratic) of the relationship between HRV and positive affect (including state/trait self-compassion). Importantly, like many ongoing and recently published tVNS studies, the current study protocol and analysis plan have been pre-registered (https://osf.io/4t9ha). In addition, as a point of clarification, while the study is registered as a clinical trial (ClinicalTrials.gov, Identifier: NCT05441774) the aims are *not* to examine the safety, efficacy or effectiveness of a treatment for a specific condition, nor to establish a ‘treatment dose’.

## Materials and methods

The study received institutional review board approval (University College London Research Ethics Committee; study reference number 0760/006; approval date: 11 May 2021).

### Design

This is a proof-of-concept, experimental-medicine study that examines the separate and combined effects of active auricular tVNS and compassion-focused imagery using a parsimonious 2 x 2 between-subjects factorial design, with repeated assessment of outcomes. The between-subjects factors are *stimulation condition* (active versus sham) and *imagery condition* (compassionate imagery versus sham imagery). Thus, participants are assigned randomly to one of four conditions (n = 30/condition):

- Sham (earlobe) stimulation + sham imagery (draw-a-face-in-imagination)
- Active (tragus) stimulation + sham imagery
- Sham stimulation + Active (compassion-focused) imagery
- Active stimulation + Active imagery

**Fig 1:**
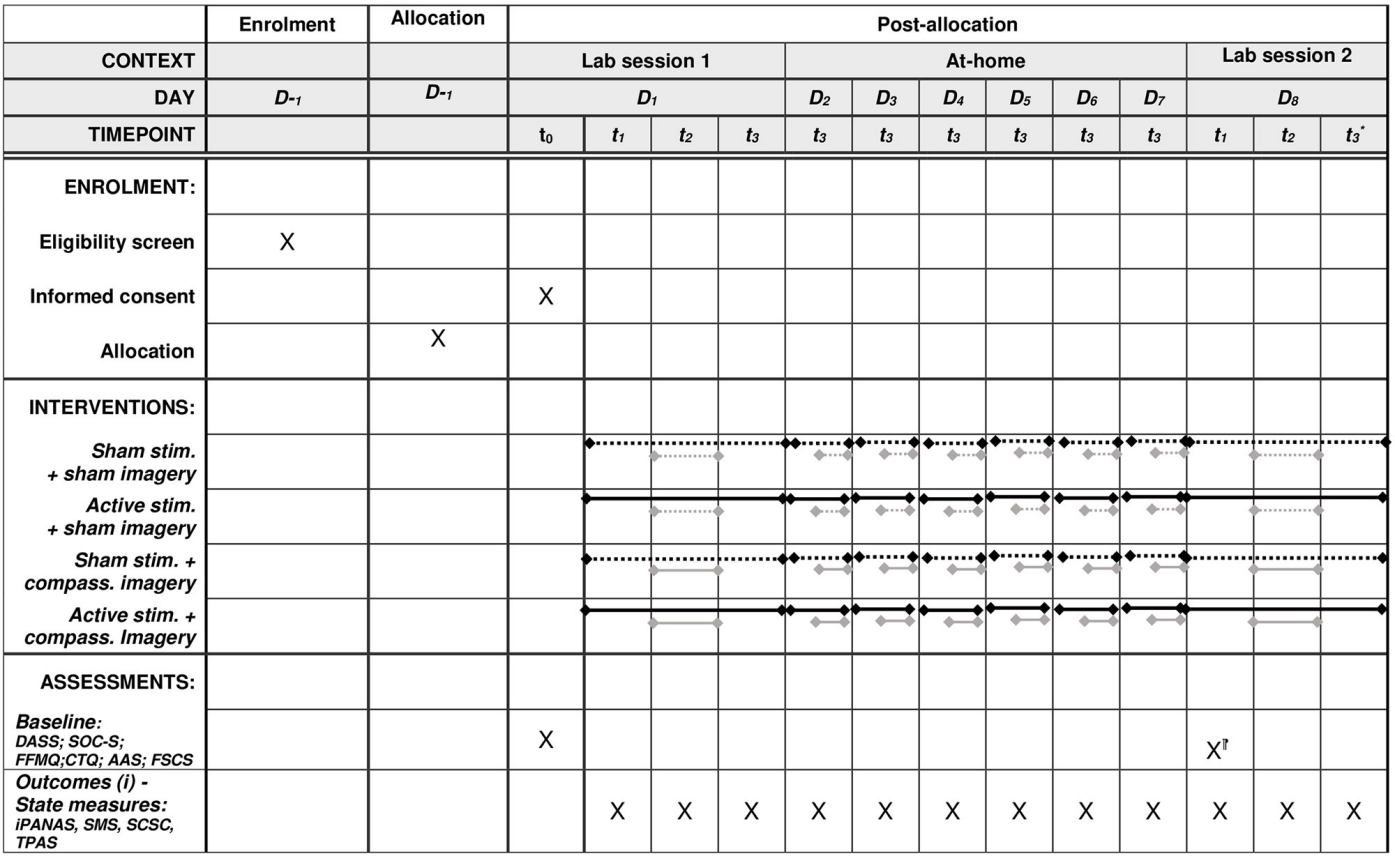
Standard protocol items: Recommendations for Interventional Trials (SPIRIT) schedule of enrolment, interventions, and assessments.

Participants attend two in-person lab sessions separated by one-week (Session 1/Day 1; Session 2/Day 8), during which psychophysiological (eye-tracking and HRV) measures are taken, alongside self-report measures of subjective state (see procedure description below). In addition, participants are instructed to use the tVNS device according to the condition (active versus sham) to which they were randomly assigned on Session 1, alongside their assigned imagery task at home for six sessions, between Sessions 1 and 2 (i.e. Days 2–7), during which they repeat the same subjective state measures used in the lab sessions (Fig 1; Standard Protocol Items: Recommendations for Interventional Trials, SPIRIT).

**Fig 2:**
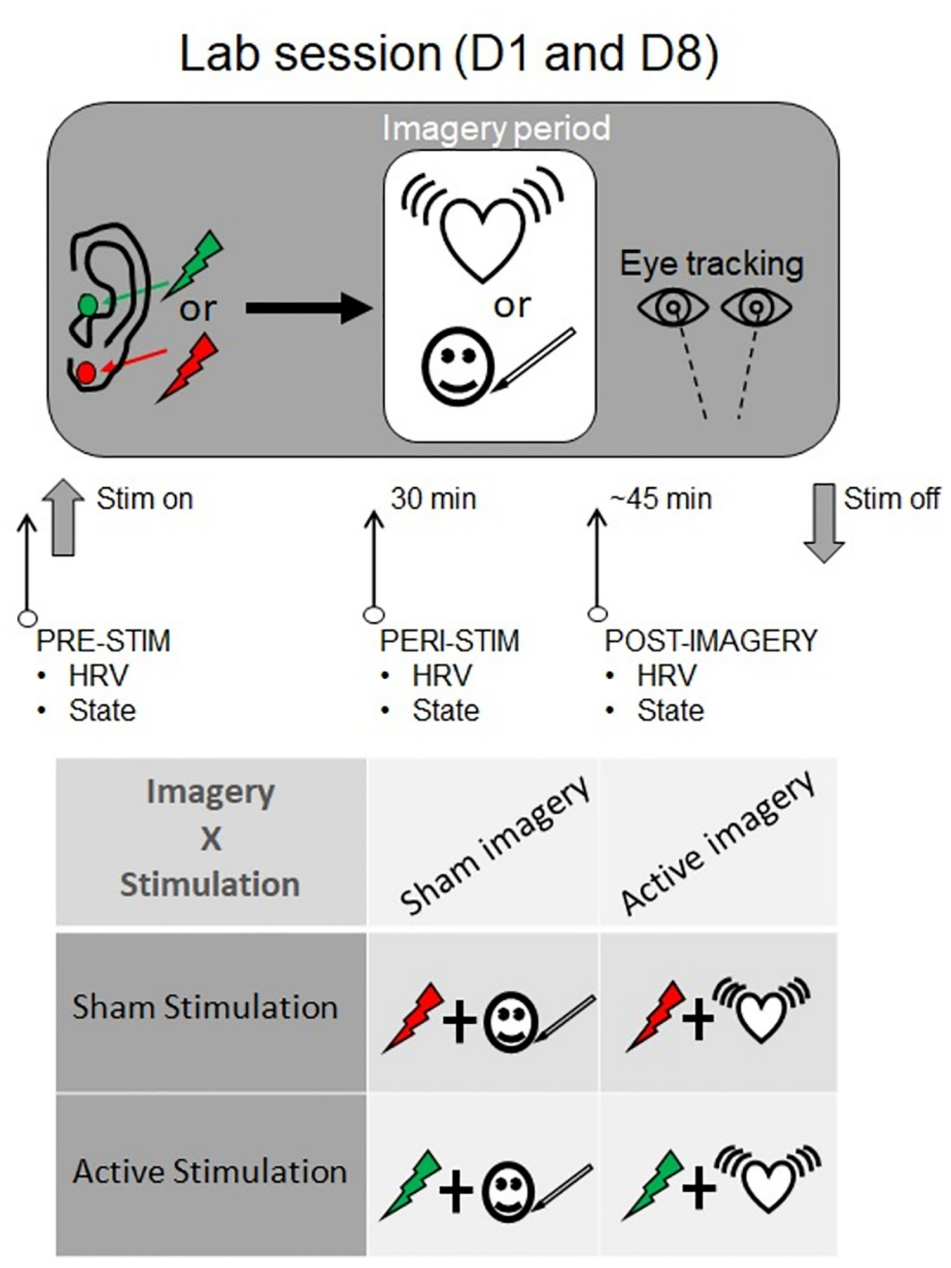
Procedural sequence for the two lab sessions (top panel). PRE-STIM refers to the assessment period immediately before the stimulator is switched on. PERI-STIM (before the imagery tasks) assessments occur after 30 min of continuous stimulation in the absence of any concurrent task. POST-IMAGERY measures are taken ~15 min after the PERI-STIM measures on lab Session 1 (Day 1) and ~10 min after on Session 2 (Day 8). The different timings are a result of the briefer recorded instructions used on Session 2 (i.e. only section 3 of the audio imagery instructions is played to participants; see details under *Mental Imagery Tasks*). The block of ‘State’ measures at the three timepoints consists of the iPANAS, SCSC Scale, 5-items from the SMS, TPAS and VFIQ (see text for details). The bottom panel illustrates the 2 x 2 design and the four conditions used in the study. Participants complete the same stimulation and imagery procedures on both lab sessions (and during the intervening at-home sessions).

As such, the within-subjects factor of ‘*time-point*’ will be used to analyse acute (within-session) effects of the stimulation and imagery manipulations (pre-stimulation, peri-stimulation; post-imagery; Fig 2), and the separate within-subjects factor of ‘*day*’, will be employed in the analyses of sustained (between-session) effects.

### Study setting

This is a single-center, laboratory-based study conducted in the Clinical Psychopharmacology Unit at University College London, UK.

### Participants

In line with experimental medicine study design standards, participants are non-clinical (healthy) volunteers, recruited from University College London and surrounding areas using online adverts (e.g. Call For Participants). They receive £80 for taking part. Written consent is obtained and recorded electronically before any experimental procedure is performed.

A target sample size of n = 120 was deemed to be feasible given our resource constraints. A sensitivity analysis was conducted using the pwr2ppl package in R [46] with a variety of simulated mean values on the Self-Compassion-Self-Criticism (SCSC; [38]) scale. This indicated that for the final within-lab-session timepoint (post-imagery), a mean difference of ≥11.1 points (common SD = 15) between the double-sham and double-active conditions on either subscale of the SCSC could be detected using independent samples t-tests with 80% power (two-sided; α = 0.05) with group sizes of n = 30. Although an average difference of 11.1 points on the SCSC scale represents a relatively large effect size (SMD = 0.74), it is in line with group differences reported in previous studies using the self-compassion subscale of the SCSC scale following a variety of biological and behavioral manipulations [42–44]. Larger effects (reductions) on the self-criticism subscale were reported in these studies [42–44]. As such, the design may have greater power to detect between-group effects on self-criticism. A sample size of n = 60 per (stimulation) condition is also adequate to detect within-condition changes in either SCSC subscale of ≥ 5.6 points (standardized mean difference = 0.37) between pre-stimulation and peri-stimulation or between pre-stimulation and post-imagery timepoints collapsed across imagery conditions (based on two-sided, paired-samples t-tests). Of course, the strength of conclusions derived from such within-condition comparisons will be lower than between-group effects.

Inclusion criteria: participants are required to be 18–35 years old, fluent in English and have good (including corrected) vision and hearing. Exclusion criteria: participants are not included if they have any of the following characteristics: current use of any medication for a psychiatric condition, regular use of any medication used to treat a cardiovascular or inflammatory condition, use of any illicit recreational drug >2/week, regular weekly consumption >14 standard UK servings (’units’) of alcohol. Additional exclusions are: current treatment for any mental health condition, scores on screening measures of depression (Patient Health Questionnaire; 2-item version [47]) or anxiety (Generalized Anxiety Disorder rating scale, 2-item version [48]) indicating significant levels of anxiety or depression (scores on either >4), history of serious mental health problems, past or current cardiovascular disease or neurological problems, current/past chronic/recurrent facial or ear pain, skin irritation/broken skin at the stimulation site, (likelihood of) pregnancy during study, and previous adverse response to meditation. Participants self-declare an absence of these conditions during screening.

### Interventions

#### Transcutaneous vagus nerve stimulation

In line with minimum reporting standards for tVNS studies [49], stimulation parameters are described in Table 1. Sham stimulation of the earlobe and active stimulation of the tragus is delivered using a Parasym tVNS device (Parasym Ltd, United Kingdom). The pulse width, frequency and other parameters used here are similar to those reported in the majority of studies on cognitive effects of tVNS (see [50]). For each stimulation session, participants are instructed to increase the current level from 0 mA in one-unit increments on the tVNS device (corresponding to steps of 0.8 mA per increment, based on a fixed resistance of 500 Ohms). The stimulation level used for each participant is set separately on each of the eight lab/at-home sessions such that it exceeds the participant’s sensory threshold (the point at which they feel a clear ‘tingling’ or pulsing sensation that is not painful). Because skin resistivity can change within a stimulation session (e.g. due to changes in moisture or slight changes in the position of the electrodes), participants are instructed to adjust the stimulation level appropriately during each lab/at-home sessions such that it remains comfortably above threshold level. Stimulation intensity on the device (range 0–40) is recorded during the session.

**Table 1:** Stimulation parameters in accordance with minimum reporting standards.

Stimulation parameter	Details
Device manufacturer	Parasym Ltd
Control	Variable current (constant voltage in tissue)
Individualization	Individualized intensity, above sensory threshold.
Direction	Preferentially afferent ^I^
Location	Left earlobe; left tragus (anterior and posterior surface)
Electrode	
• Number	Two
• Composition	Gold plated
• Attachment method	Ear-clip
Duty cycle	Constant (no on/off cycling)
Frequency	20 Hz
Pulse shape	Rectangular
Pulse width	200 uS
Amplitude	0.8 mA/increment (based on a fixed resistance of 500 Ω) ^I^
Stimulation period	
• Session 1 (Day 1)	~70 min*
• Days 2–7	~30 min/day
• Session 2 (Day 8)	~65 min^⁋^
• Total (intended)	~315 min^±^
Timing relative to task	Offline (i.e. pre-imagery) and online (peri-imagery and eye-tracking)

^I^ Information provided by manufacturer

* 45 min pre-stimulation + peri-imagery period plus ~25 min until end of eye-tracking.

^⁋^ 40 min pre-stimulation + peri-imagery period plus ~25 min until end of eye-tracking.

^±^ 315 min represents the maximum total stimulation over the course of 8 days. The expected range is 135 min (100% compliance with the two in-session stimulation protocols + 0% compliance with at-home stimulation) and 315 min (100% compliance with the two in-session stimulation protocols + 100% compliance with at-home stimulation)

An initial (pre-imagery, ‘offline’) 30-min active/sham stimulation period begins immediately after the block of pre-stimulation state measures. During this 30 min period of stimulation, participants are asked to relax and watch scenes from a nature documentary (Blue Planet II, 2017) which were selected to be emotionally neutral. At the 30 min mark, the imagery instructions are presented (see below) and stimulation continues throughout the imagery task (‘online’ stimulation). Approximate total stimulation periods for the lab and at-home sessions are outlined in Table 1.

#### Mental imagery tasks

*Imagery task design*. As noted above, two sets of imagery instructions are used in this study: compassion-focused imagery (the active imagery condition) and ‘draw-a-face-in-imagination’ (the sham imagery condition; also referred to below as the ‘draw-a-face task’). The instructions in the two conditions were designed to be well-matched on various attributes: number of words (compassion: 1321 words, draw-a-face: 1356 words), complexity of language (Flesch-Kincaid grade level—compassion: 10.5; draw-a-face: 8.4) and total length of the audio recording (compassionate imagery: ~16 min; draw-a-face ~15 min). This matching was achieved after extensive editing and feedback from clinician-researchers (n = 11) who had no other involvement in the study other than providing feedback and rating the introductory aims/rationale part of the imagery/tVNS instructions on dimensions of (i) compellingness of the explanations, (ii) credibility of the treatments and (iii) extent to which participants might expect to experience enhancement of imagery capabilities as a result of using tVNS. The ratings provided by researchers were approximately equivalent, with averages for the three items ranging from 5.2 to 5.8 (out of 7) in both imagery conditions.

*Imagery task deployment in the experiment*. The finalized instructions used in the actual study are presented after the initial task-free (off-line) period of stimulation while (online) stimulation continues. For both imagery conditions, the instructions are presented as audio recordings separated into three sections during Session 1 (all audio was recorded using the same male voice). The first, introductory audio (referred to in the *Imagery task design* paragraph above) provides a description of the aims of the specific imagery task to which the participant was randomly assigned (compassion versus draw-a-face) and the rationale for combining mental imagery with tVNS. The second section provides an orientation to and more specific description of the respective imagery tasks, and the third section is the actual guided imagery task that participants actively engage in. The first introductory audio instructions state either that tVNS might be “…an effective non-invasive way of activating brain processes involved in producing feelings of safeness and comfort, which are a prerequisite for self-compassion” (compassion condition) *or* that tVNS could be “…an effective non-invasive way of activating brain processes involved in forming and manipulating mental images” (draw-a-face condition). These instructions also inform participants that stimulating the VN with tVNS “might make it easier to form ….mental images”. Additionally, they state that we hope our findings will enable new treatment techniques to be developed either “for people with depression and other psychological disorders, in which self-compassion is often lacking” (compassion imagery) or “for improving memory for faces that would otherwise be difficult to remember… [for] application in forensic cases, for example” (draw-a-face imagery). We have already outlined the theoretical basis for a potential enhancement of (the effects of) compassionate imagery by tVNS in the introduction, and this formed the basis for the simplified and abbreviated explanation provided to participants (above). However, it was also important to provide a similarly credible explanation to participants in the control/sham draw-a-face task condition in order to ensure similar (positive) expectations in both imagery conditions. This was deemed to be especially important for motivating participants to complete the at-home imagery tasks at an equivalent rate for the two imagery conditions (i.e. to avoid poor compliance that might be associated with a non-credible control imagery task). To determine the achieved degree of equivalence, participants provide ratings on measures of credibility/expectancy [51] on Session 1 after the introductory audio.

The third audio section for the compassionate mental imagery condition was derived from Gilbert’s ‘compassionate mind training’ [8] and adapted from previous studies [42, 43]. This instructs participants on how to direct compassionate feelings inwards, towards the self (self-compassion) using mental imagery. To avoid additional respiration-mediated effects on vagal activity, and to enable close matching with the sham/control imagery condition on non-imagery aspects, the compassion imagery instructions used here do not contain a paced breathing component (*c*.*f*. [42, 43]). They also deliberately exclude a focus on grounding/posture and mindfulness (e.g. mindful responding to self-criticism).

The third audio section for the sham draw-a-face task, consists of instructions to imagine using a drawing/painting implement of the participant’s choice to recreate an unfamiliar face in imagination. To provide a standard stimulus for this imagery condition, participants complete a ‘face rating task’ at the start of Session 1. It should be reiterated that this procedure is part of the control imagery condition, and that the elaborate task demands described below were simply designed to enhance the credibility of the draw-a-face task, and to increase the plausibility of the explanation that tVNS could improve mental imagery ability, and hence memory for faces. For the face rating task, participants are presented with an unfamiliar face in the form of a computer-generated photograph, which they rate on a number of characteristics (see details on the face rating task in the Incidental and control self-report measures, below). The instructions ask participants to recall the face of the individual from the face rating task and then to “add details to your imaginary canvas” as different parts of the face are mentioned in the audio instructions. Participants are re-presented with the original unfamiliar face/photograph from the face rating task after completing the draw-a-face task, and the post-imagery state measures, and rate its resemblance to the mental image they formed during the draw-a-face task. They are also asked to memorize the photograph, so that they will be able to repeat the draw-a-face task during the at-home sessions in the absence of further presentations of the photograph.

On lab Session 2 (Day 8) participants only listen to the third recording (the guided imagery task). An abbreviated version of the third recorded section is also used in the at-home sessions on Days 2–7, the audio for which is presented via Qualtrics. The latter is also used to register the duration of participants’ engagement with imagery instructions during the at-home imagery sessions. Verbatim scripts for the two imagery conditions are available from the corresponding author.

### Measures

#### Self-report outcome measures

To limit the chances of data entry errors, all self-report measures are presented (and participant responses recorded), via the online survey program, Qualtrics. State measures are taken at three time-points on Session 1 and Session 2: before stimulation, during stimulation and after the imagery task (as outlined in Figs 1 and 2: t_1_ = *PRE-STIM*; t_2_ = *PERI-STIM*, t_3_ = *POST-IMAGERY*). Each block of state measures consists of: International (short-form) Positive and Negative Affect Schedule (iPANAS, [52]), Self-Compassion-Self-Criticism (SCSC) Scale [38], five items from the State Mindfulness Scale (SMS [53]) as used in Shoham et al’s experience sampling study and Types of Positive Affect Scale (TPAS, [54]). The block of state measures concludes with the ‘Vividness of Facial Imagery Questionnaire’ (VFIQ, a sham measure; see below). These state measures are also administered post-imagery on Days 2–7 (denoted by ‘t_3_’ in Fig 1; note, they are only administered once on Days 2–7 in order to minimize participant burden). Of these, subscales of the SCSC are considered the primary self-reported outcomes (in addition to HRV measures, below), and iPANAS, TPAS (safe-content) and SMS items as secondary.

#### Incidental and control self-report measures

On Session 1, participants provide demographic details (age, sex, ethnicity, years of education, use of hormone-based contraceptives and menstrual phase) and, if relevant, details on any mind-body practices they use (type, regularity, duration per session and history of practice). They complete the following questionnaires intended to tap relatively stable psychological characteristics or ‘traits’ in the following order on Session 1: Depression, Anxiety and Stress Scale (DASS-21) [55], Sussex-Oxford Compassion Scale (Self version; SOC-S [37]), Five Facet Mindfulness Scale (FFMQ-15 [56]), Childhood Trauma Questionnaire (CTQ-SF; short-form [57]), Adult Attachment Scale (AAS; revised, close relationship version [58]), and Fear of Self-Compassion Scale (FSCS [59]). Of these, the DASS-21, SOC-S and FFMQ-15 are repeated on Session 2 (Day 8). These stable/trait measures are intended to characterize/describe the participant sample and might be examined as moderators of the effects of stimulation/imagery condition.

A face rating task is included in order to provide a standardized stimulus for use in the draw-a-face task. As noted under the ‘Mental Imagery’ section above, participants view a computer-generated photograph of an individual (https://generated.photos/) and are asked to examine the face carefully while rating it for friendliness, approachability and trustworthiness (1 = not at all; 9 = very). These ratings are entirely incidental and are included only to encourage relatively deep encoding of the face stimulus so that participants randomized to the draw-a-face condition can more easily recall the face when asked to bring it to mind during the draw-a-face task. Note however, that while the rating task is only relevant for participants in the draw-a-face condition, the ratings are completed by all participants—including those randomized to the compassionate imagery condition—in order to match the groups for completed tasks as closely as possible.

Relatedly, to increase the credibility of the draw-a-face imagery task, and of the explanation that tVNS might enhance “brain processes involved in forming and manipulating mental images”, a sham outcome, the VFIQ was devised to assess (simultaneously with the other primary and secondary state outcome measures described above) participants’ capacity for vivid facial imagery. Again, this is completed by all participants and consists of ratings of vividness of current mental imagery of the faces of three people: a relative/friend, best friend from primary school, and the individual in the photograph from the face rating task above (1 = not at all; 7 = very). Again, these are sham ratings and are not expected to be affected by tVNS.

Three credibility/expectancy questions are included after the first (introductory) audio section on Session 1, each rated on a 9-point Likert scale relating to (i) how logical the use of tVNS to improve mental imagery seems, (ii) how successful it will be in changing mental imagery abilities, and (iii) how likely the participant would be to use tVNS to improve your mental imagery abilities?

Compliance with, and response to instructions are assessed after the imagery task on each session (including Days 2–7) using a series of questions that assess (i) how closely the audio imagery instructions were followed, (ii) clarity of mental images during the imagery tasks, (iii) ease of forming these mental images and (iv) affective response to these mental images, using 9-point Likert rating scales. In addition, participants rate the extent to which they believed their ability to form mental images improved as a result of tVNS. Also, in relation to compliance, participants are encouraged to indicate the extent to which they complete the at-home tasks. Thus, at the end of Session 2, they receive these instructions: “*Although we encouraged you to complete the at-home tasks every day*, *we do understand that this can be difficult for people*, *and that it’s sometimes impossible to do these tasks every day*, *or even on most days*. *Now that you’ve completed the study*, *it’s very important for us to know how many days you actually completed the stimulation and how many days you followed the imagery instructions*. *We can then factor this into our statistical analysis and get a more accurate estimate of the real effects of stimulation*. *Please answer as honestly as possible*. *Your responses will have no effect on your payment for participation*!”. They then indicate the number of days (out of 6) that they performed the stimulation, as well as the number of days (out of 6) they listened to the imagery instructions during the stimulation. Various effects of stimulation are also assessed using 9-point Likert scales in relation to physical sensation (tingling, pulsing, discomfort) and adverse effects are recorded for ear pain, face pain, headache, skin irritation, dizziness, upset stomach, common cold symptoms, cough and ‘other’.

#### Psychophysiology

A heart-rate monitoring device (Bodyguard 2, Firstbeat, Finland) with a sampling rate of 1kHz and a modified lead II configuration (electrodes placed below the left clavicle and right ribcage; no ground electrode) is used to record interbeat (RR) interval data throughout Sessions 1 and 2. The Firstbeat Bodyguard device has been validated against gold standard laboratory-based ECG recording procedures [60]. During offline analysis of HRV data, RR-time plots will be visually inspected and, if necessary, artifact corrected using the software package, Kubios [61]. The latter software will also be used to extract time and frequency domain HRV metrics for the relevant 5-min recording periods (see below).

There is no single widely used HRV metric in published studies of tVNS [62] or compassion-induction procedures [24]. Indeed, HRV analysis software typically provide numerous output metrics, which in the absence of pre-registration, potentially introduce large amounts of researcher degrees of freedom. Our analysis will therefore focus *a priori* on two HRV measures that are putative measures of ‘vagal tone’: the time domain measure, root mean square of successive differences (RMSSD) between normal heartbeats, and the frequency domain measure, high frequency (0.15–0.4 Hz) power [63, 64], which will be determined via fast Fourier transform. Any analysis reported on other HRV metrics will be considered exploratory. HRV data will be extracted from three continuous 5-min recordings corresponding to the three self-report assessment timepoints. Specifically, pre-stimulation HRV metrics will be obtained from the RR data during the 5-min immediately before the start of the stimulation procedure; peri-stimulation HRV corresponds to the 5-min of stimulation before the start of the imagery procedure and the third period (peri-imagery), the last 5-min of the guided imagery task.

To assess participants’ oculomotor attentional bias towards compassionate faces, an eye-tracking computer task is deployed on an eye tracker with a 1kHz sampling rate (EyeLink 1000, SR Research, Canada). The tracking camera is placed approximately 60 cm from participants’ gaze position, which is held constant by a chinrest. The stimuli (450 x 430 pixels) are presented on a 17 inch, 1280 x 1084 resolution monitor and consist of the neutral/compassionate faces created by Falconer and colleagues [39]. The stimuli are formed of six different composite individuals (‘identities’; see ref. 39), each of which has five expressions differing in the extent to which it has been morphed between one extreme (0% compassion; neutral) and another (100% compassion). In the task, participants view two horizontally adjacent faces, appearing simultaneously. Each pair includes one face which has not been morphed (neutral) and one that has been morphed towards the compassion prototype at 25%, 50%, 75% or 100%. For each of the 6 identities, the 0–25, 0–50, and 0–75 pairs appear 4 times, whilst the high contrast pairs (0–100) appear 8 times. This gives a total of 120 trials. The stimuli are balanced such that the compassion face appears on each side of the screen an equal number of times. The trials appear in a randomized order. Participants view the faces for 3000ms before being promoted to respond via keyboard response (left or right) to the face they prefer. Measures of interest are dwell time, first fixation and pupil size as oculomotor indices of attentional bias and arousal during stimulus viewing. We are not aware of previous research employing eye-tracking measures on compassionate face stimuli, and this approach is used here in an exploratory manner. Preference and reaction times are recorded incidentally.

#### Randomization

Random assignment to the four conditions was achieved using a random number generator (https://www.random.org/sequences/). Unique, non-repeating sequences of numbers in three blocks: 1–40, 41–80, 81–120, each corresponding to a participant id were obtained in a randomly ordered sequence. In unordered form, each id was assigned a number from 1 to 4, corresponding to one of the experimental conditions, in a repeating sequence (1,2,3,4,1,2,3,4, …etc). As such, upon reordering (from 1 to 120), participants were randomly and evenly assigned to the four experimental conditions. The sequence was generated internally by the research team. Enrollment and assignment is handled internally, by the research team.

#### Blinding

The effects observed in studies of neurostimulation may, in part, be driven by expectancy and prior beliefs [65]. The aims and hypotheses of the study are therefore concealed from participants. In particular, participants are not made aware of the number and nature of experimental conditions. Study information provided to participants prior to Session 1 describes tVNS, but conceals the nature of active versus sham stimulation. Study information also refers to a ‘mental imagery task’, but not the exact form such a task might take. The nature of participants’ imagery condition (compass/active versus draw-a-face/sham imagery) is only disclosed prior to consent on Session 1. Participants are debriefed fully at the end of the study.

### Procedure

#### Screening

Participants responding to adverts for the study are directed to a site describing basic study information. If interested, they undergo the first (online) step in the screening process. This is intended to ensure basic inclusion and exclusion criteria are met. Those meeting basic criteria undergo a follow-up telephone screening, which occurs after the participant has received and read the study information sheet. This telephone screening aims to (i) verify responses to the online screening and (ii) establish an initial rapport with the participant and ensure they are aware of the study requirements.

#### Session 1 (Day 1)

Upon arrival, participants confirm they have read the study information sheet and are provided with additional standardized information on the nature of the imagery task to which they have been randomized. Formal consent is sought only after this additional information is provided and participants have been given an opportunity to ask questions.

The heart rate monitoring device is attached at the beginning of the session to allow sufficient time for signal stabilization before the first HRV sampling period. Participants then provide details about demographics and their use of mind-body practices. After the face-rating task, they complete the trait measures. The tVNS electrode ear-clip is attached, but not switched on, after completion of the SOC-S (~10 min before the start of the first block of state measures, prior to stimulation). This ~15 min unstimulated run-in period is intended to allow participants to become accustomed to the sensation of the ear-clip, and desensitize to any unfamiliar sensations or minor discomfort before the pre-stimulation state measures are taken. At this point, the researcher explains briefly how the ear-clip cable is connected to the device and ensures that the participant understands how to minimize the risk of the ear-clip becoming detached during the session, and how to reattach it if required.

To orient participants to the correct timeframe for the state measures, the researcher explains that the upcoming block of measures assesses how the participant is feeling “*right now*” (the exception is the SMS which enquires about “*the last 5 minutes*”). After these pre-stimulation state measures, the stimulation procedure commences; after 30 min of continuous stimulation, participants complete the block of peri-stimulation state measures, which is identical to the pre-stimulation block. This is followed by the imagery procedure, which incorporates ratings of expectancy and credibility. The final state measures (post-imagery; Figs 1 and 2) are repeated straight after the imagery task.

The final substantive task on Session 1 is the compassionate face eye-tracking task. Before the session ends, participants are asked to honestly rate their compliance with the imagery instructions.

#### Session 2 (Day 8)

The second lab session is largely identical to Session 1, with the exception that the AAS, CTQ-SF and FSCS scales are not repeated and the imagery procedure is abbreviated, starting at the third recorded section (the guided imagery instructions), omitting the explanation/orienting sections.

#### At-home sessions (Days 2–7)

Participants log on to the study website once a day for the six at-home sessions between the two lab sessions. As far as possible, they are instructed to complete the at-home stimulation/imagery tasks at the same time each day. Participants’ use of the study survey website (e.g. frequency and duration of interaction with the site) is automatically recorded, providing an indirect measure of compliance/engagement (with imagery instructions). In addition, all participants’ activity on the website is monitored. If they fail to logon for ≥2 days, a member of the research team telephones the participant to request completion of tasks/measures on subsequent days. Similarly if the internal timer on the survey program indicates that a participant might have skipped ahead, past the imagery instructions on ≥2 occasions, a researcher calls to ensure that they have understood the requirements of the task and request that this step is completed on subsequent days.

### Statistical analysis

Assuming that the usual assumptions of linear modelling are met, linear mixed models (LMMs) will be used to analyze repeated measures data with >2 timepoints. LMMs are appropriate for modelling the serial dependence in outcome from the same participant and are more effective at handling missing data than analysis of variance. Random intercepts per participant will be included in models and the effects of including interaction terms and random (Timepoint or Day) slope terms will be tested. AIC and BIC minimization will be used as criteria for model selection. Univariate analyses of variance will be used to test imagery/stimulation effects at the post-imagery timepoint for the lab sessions. Specific tests of hypotheses will be performed using independent- or paired-samples t-tests of outcomes. If assumptions for linear modelling are not met (e.g. in cases of serious violations of distributional assumptions as indicated by visual inspection of QQ plots), generalized linear mixed models will be used with appropriate distribution and link functions. This analysis approach will be applied to the primary outcomes: SCSC subscales, and HRV (RMSSD and HF power), as well as the secondary subjective measures that might be responsive to active stimulation and/or compassionate imagery including the safe/content subscale of the TPAS, iPANAS-positive affect (effects on iPANAS-negative will also be assessed in exploratory analyses) and SMS 5-item total. These secondary subjective measures will also be taken pre-stimulation, peri-stimulation and post-imagery on lab Sessions 1 and 2 (Day 1 and 8) and post-imagery on Days 2–7, as for the primary measures.

Analysis of eyetracking metrics will involve comparing neutral expressions with high intensity (100%) compassion expressions, with Stimulation and Imagery condition as independent variables. Additional analyses will include ‘Intensity’ (0, 25, 50, 75, 100%) as an additional explanatory variable.

Two-tailed tests will be used, with a p value threshold of ≤0.05. The latter might be considered liberal in light of the number of pre-specified primary outcomes (self-compassion, self-criticism, RMSSD and HF power). However, given the preliminary nature of the study and the risk of type II errors, no downward adjustment to the α level was pre-specified.

Further details on analyses, including exploratory approaches are described on the Open Science Framework (https://osf.io/4t9ha).

## Discussion

We describe a protocol for a proof-of-concept study that tests the effects of both off- and on-line noninvasive stimulation of the VN on a specific, positively-valenced cognitive-affective state, namely (self-) compassion, and related outcomes. As such we are testing the sufficiency of VN activation via tVNS for generating compassionate responding and, more plausibly perhaps, whether vagal activation via tVNS provides a permissive (central and/or peripheral) physiological context within which such a state could arise [20]. A demonstration of effects indicative of tVNS-induced enhancement of compassionate imagery would provide support for a causal role of the VN in compassionate responding. Moreover, such a finding would have promising implications for the use of such bioelectronic strategies as adjuncts to compassion-focused treatments for psychopathology.

Although we know of no studies that have examined the effects of tVNS on compassionate responding, related research using transcranial direct current stimulation (tDCS) has shown enhanced heart rate variability (via putative neurocardiac coupling, [66]), and increased compassion-related (‘soothing’) positive affect during stimulation [67]. However, these studies have tended to test offline stimulation (i.e. in the absence of a relevant concurrent task) or in the context of a physical or psychological challenge (e.g. stress induction). By contrast, a strength of the current protocol is that there is a clear—though as yet, relatively untested—theoretical rationale [10, 19–21, 68] for testing interactive effects between active tVNS and an active cognitive-affective task whose performance is thought to partly rely upon vagal activity. Moreover, our use of repeated stimulation episodes allows us to test whether the effects of stimulation accumulate through ‘use dependence’ [69]. To the extent that practicing self-compassion involves learning, and that enhanced vagal activity encourages adaptive plasticity [45], we can expect the effects of active stimulation to be cumulative when repeatedly paired with self-compassion ‘training’. Such cumulative effects would likely be most evident in those who practice most regularly (i.e. are most compliant with the at-home stimulation/imagery tasks).

This remains a relatively contentious area of science [70], with mixed findings in relation to biomarkers of vagal activity in response to tVNS [62], and limited high quality empirical evidence for a role for the VN in compassionate responding [23]. Our data handling and analysis plans have therefore been pre-registered on the Open Science Framework (https://osf.io/4t9ha). Of course, this does not preclude discovery-oriented analyses of moderators or sub-groups (e.g. those showing increases in HRV during stimulation versus those who are ‘non-responsive’ or those showing varying degrees of ‘homework’ compliance), although any such exploratory analysis would be clearly described as such and any resulting ‘positive findings’, appropriately caveated.

### Limitations of the study design

There will likely be unknown/unexplained sources of variance in our data that could substantially affect our ability to detect the effects of tVNS. For example, there appears to be substantial individual anatomical variability in vagal innervation of the tragus, and in the number of myelinated axons in the auricular branch of the VN. An absence of reliable effects of tVNS, particularly on the positive control variables, namely HRV, might reflect ineffective stimulation of the VN in some individuals. While a number of studies have shown that active tVNS increases HRV [71–73], other researchers have raised doubts about whether increased HRV is indeed a reliable indicator of successful stimulation [62, 74] (in fact some researchers even doubt that HRV reflects vagal tone: [75]). Part of the difficulty in establishing reliable biomarkers of tVNS efficacy is the inordinately large number of combinations of stimulation parameters that might affect successful stimulation, and by extension, HRV.

A strength of many published tVNS studies is their use cross-over designs. Although such within-subjects designs are desirable from a statistical noise-reduction perspective and are feasible if all conditions can be performed within a single or small number of session(s), our use of repeated sessions of stimulation and imagery makes it highly impractical to employ a cross-over design. In particular, it would be very challenging to recruit and retain participants to perform daily tasks that would become highly repetitive and burdensome over the required period. As such, on balance, we decided that a between-subjects design was necessary, although this is only powered to detect relatively large between-group effects.

Our design is based on the assumption that the stimulation location, and other stimulation parameters, as well as our selection of measured variables are the ‘correct’ ones for generating and detecting the effects of tVNS. It is possible that slightly different stimulation parameters or targeting the cymba conchae or cervical branch of the VN would be more effective. Indeed, even supposing that the tragus is the optimal locus of stimulation, inter-individual anatomical variability in vagal innervation [16], might contribute to sufficient statistical noise to obscure group level average differences. Our current state of knowledge is too limited to have high confidence that any single set of parameters is optimal for any particular cognitive-affective task, let alone for enhancing compassion, for which there is no precedent in the existing literature. We therefore use the manufacturer default settings of the tVNS device, which in one study were shown to increase HRV [72], and have been found to have therapeutic effects in clinical trials, though on unrelated outcomes (e.g. [76]). We believe that this is at least a reasonable starting point for testing the effects of interest.

Unlike the increasing use of double blinding in tDCS studies, it is still common for tVNS studies to be single-blind. Although researchers’ influence on participants’ responses is minimized by the limited and scripted nature of their interaction with participants in the current study, the researchers in the current study are not blind to treatment allocation. Moreover, although the study aims are nominally masked from participants, there was no way within our design to protect against curious and diligent participants from learning about the nature of active versus sham stimulation, and about the nature of the experiment as a whole.

These limitations notwithstanding, we believe the study protocol provides an opportunity to test the direct, causal role of the VN in modulating compassionate responding. Even if effects are relatively small (and statistically non-significant) but nonetheless point to a possible additive/synergistic effect between compassionate imagery and active stimulation at a descriptive level (i.e. largest effects on self-compassion/self-criticism or other outcomes in the active tVNS + active/compassionate imagery condition), this would still provide valuable information (e.g. likely effect size; informativeness of control groups) for planning future related studies. Alternatively, a more convincing demonstration of synergy/additivity between the two interventions might provide the basis for preliminary clinical testing of this combination strategy, for example in participants with high levels of self-criticism/low self-compassion.

## Supporting information

S1 Checklist SPIRIT 2013 checklist: Recommended items to address in a clinical trial protocol and related documents*. (DOC)

S1 Protocol (DOCX)
