# Supplementary material for: A bioelectronic route to compassion: Rationale and study protocol for combining transcutaneous vagus nerve stimulation (tVNS) with compassionate mental imagery
Source: PLoS One. 2023 Mar 13;18(3):e0282861. doi: 10.1371/journal.pone.0282861 (PMC10010509; doi:10.1371/journal.pone.0282861)
Supplement: S1 Protocol — (DOCX) [file pone.0282861.s002.docx]

Supplementary information for Kamboj, et al A Bioelectronic Route to Compassion: protocol

| **SECTION A** |
| --- |

| \| **A1** \| \| --- \| | **Project Title:** Transcutaneous vagus nerve stimulation (tVNS) and mental imagery | |
| --- | --- | --- | --- |
|  | Date of Submission: 02/03/2021 | Proposed Data Collection Start Date: 01/07/2021 |
|  | UCL Ethics Project ID Number: **0760/006** | Proposed Data Collection End Date: 31/12/2023 |
|  | **Is this application for continuation of a research project that already has ethical approval? *For example, a preliminary/pilot study has been completed and this is an application for a follow-up project? If yes, please provide the information requested below.*** | |
|  |  | |

| \| **A2** \| \| --- \| | **Principal Researcher**  *Please note that a student – undergraduate, postgraduate or research postgraduate cannot be the Principal Researcher for Ethics purposes.* | |
| --- | --- | --- | --- |
|  | Full Name: Sunjeev Kamboj | Position Held:  Professor of Translational Clinical Psychology |
|  | Name and Address of Department:  Clinical, Educational and Health Psychology  1-19 Torrington Place  WC1E7HB | Email: sunjeev.kamboj@ucl.ac.uk |
|  |  | Telephone: +44 (0) 20 7xxx xxxx |
|  |  | Fax: N/A |
|  | **Declaration To be Signed by the Principal Researcher**   - I have met with and advised the student on the ethical aspects of this project design *(applicable only if the Principal Researcher is not also the Applicant).* - I understand that it is a UCL requirement for both students & staff researchers to undergo Disclosure and Barring Service (DBS) Checks when working in controlled or regulated activity with children, young people or vulnerable adults. The required DBS Check Disclosure Number(s) is: **N/A** - I have obtained approval from the UCL Data Protection Officer stating that the research project is compliant with the General Data Protection Regulation 2018. My Data Protection Registration Number is: **Z6364106/2021/03/03** - I am satisfied that the research complies with current professional, departmental and university guidelines including UCL’s Risk Assessment Procedures and insurance arrangements. - I undertake to complete and submit the ‘Continuing Review Approval Form’ on an annual basis to the UCL Research Ethics Committee. - I will ensure that changes in approved research protocols are reported promptly and are not initiated without approval by the UCL Research Ethics Committee, except when necessary to eliminate apparent immediate hazards to the participant. - I will ensure that all adverse or unforeseen problems arising from the research project are reported in a timely fashion to the UCL Research Ethics Committee. - I will undertake to provide notification when the study is complete and if it fails to start or is abandoned. | |

| **SIGNATURE:** [Signed 03/03/22] | **DATE:** 03/03/2021 (additional amendments: 15.03.22 and 22.08.22) |
| --- | --- |

| \| **A4** \| \| --- \| | | **Sponsor/ Other Organisations Involved and Funding** |
| --- | --- | --- | --- |
|  |  | 1. **Sponsor:**  **UCL**  **Other institution**   If your project is sponsored by an institution other than UCL please provide details:   1. **Other Organisations**: If your study involves another organisation, please provide details. *Evidence that the relevant authority has given permission should be attached or confirmation provided that this will be available upon request.* 2. **Funding:** What are the sources of funding for this study and will the study result in financial payment or payment in kind to the department or College? *If study is funded solely by UCL this should be stated, the section should not be left blank.*   The Bial Foundation |
| **SECTION B DETAILS OF THE PROJECT** | | |

| \| **B1** \| \| --- \| | **Please provide a brief summary of the project in simple lay person’s prose outlining the intended value of the project, giving necessary scientific background.**  *(max 500 words)***.**  **Notes**   1. This study extends our recently completed study on transcutaneous vagus nerve stimulation and compassion (The effects of vagus nerve stimulation on compassionate mind training and threat processing; UCL ethics ID: 4277/001). The details below outline how this study extends that work. 2. Testing during any COVID restrictions will strictly follow SLMS GSOP relating to personal contact during the pandemic.   **Background**  This study investigates whether a form of self-affiliation, namely, **self-compassion**, can be modulated using **non-invasive stimulation** of the vagus nerve (**transcutaneous vagus nerve stimulation; tVNS**) via **the external ear** (i.e. earlobe or tragus). The vagus nerve is part of the parasympathetic nervous system (the ‘rest and digest’ system; Murray et al., 2016) and its fibres are spread throughout the upper body, face and neck, including branches that innervate parts of the external ear, close to the skin. Recent research has shown that the vagus nerve is involved in a range of psychological processes including memory, threat/fear processing and positive affect (Kirby et al, 2017; Burger et al. 2016). For example, vagus nerve activity increases after compassionate mental imagery (Kirby et al., 2017). This activity can be assessed non-invasively using an ECG device that measures changes in heart rate (heart rate variability; HRV), which is an index of parasympathetic nervous system functioning.  Previous research has examined the effects of inducing compassionate feelings and then measuring vagus nerve activity (by measuring HRV). However, no published study has demonstrated the association between vagal activity and compassion by modulating the vagus nerve itself. Such studies not only have theoretical implications, but are also potentially valuable in the future development of interventions that employ tVNS or similar technology to augment psychological treatments incorporating compassion-focused techniques (e.g. compassionate mind training; compassion focused cognitive therapy).  In this study, we aim to *directly* test the relationship between vagus nerve activity and compassionate feelings using non-invasive stimulation of the vagus nerve in healthy volunteers. Our own recently completed unpublished related study (Title: The effects of vagus nerve stimulation on compassionate mind training and threat processing; UCL ethics ID: 4277/001) attempted to do this by **comparing** the effects of **active tVNS** **combined with compassionate mental imagery** with **‘sham’ tVNS** **combined with compassionate imagery**. However, that study only used two relatively brief tVNS sessions (only one of which was ‘active’) and evidence of vagal nerve modulation was weak. According to recent research (Yap et al., 2020), our stimulation protocol may have been insufficient to generate behavioural (including subjective) changes. In addition, that study included a compassionate imagery exercise in both the active and sham tVNS conditions, thus preventing us from examining the effects of imagery in isolation.  Our **primary research questions** are therefore:  ***(1)*** Is the tVNS protocol used here (7 stimulation sessions instead of 2, as used in our previous study) associated with reliable physiological (e.g. HRV) changes between lab Sessions 1 and 2?  ***(2)*** Is tVNS sufficient to **generate** compassionate subjective states; are these effects cumulative?  ***(3)*** Does tVNS **augment** the response to compassionate mental imagery compared to control imagery?  Because we are interested in the **interaction between stimulation and the mental imagery task**, we will use a **four group factorial design** in which participants are randomly assigned to **(i) tVNS + control imagery**, **(ii)** **tVNS + compassionate imagery**, **(iii)** **sham-stimulation + control imagery** and **(iv) sham-stimulation + compassionate imagery**. This design will help us establish whether tVNS alone generates compassionate behaviours/feelings and therefore allow us to directly link the effects of activity of the vagus nerve to compassionate feelings. Alternatively, the effects of tVNS on compassionate behaviour(feelings) may only be observed when combined with compassionate imagery (i.e. priming the vagus nerve may enhance the effects of compassionate imagery).  **Secondary questions** relate to:   1. The effects of tVNS and compassionate imagery on recognition of compassionate versus neutral emotional expressions 2. Moderation of effects by trait compassion, mindfulness and historical trauma (these are exploratory analyses intended for hypothesis generation, and will be declared as such in pre-registration documentation). |
| --- | --- | --- |

| \| **B2** \| \| --- \| | **Briefly characterise in simple lay person’s prose the research protocol, type of procedure and/or research methodology (e.g. observational, survey research, experimental). Give details of any samples or measurements to be taken** *(max 500 words).*  **Design**  This is a randomised, controlled experimental study.  **Participants**  Participants will be healthy young volunteers.  **Screening**  Participants will respond to study advertisements which provide a link to study information and basic screening questions (age, sex, confirmation of absence of physical and mental health problems as well as the PHQ-2 and GAD-2 questionnaires to screen for severe anxiety or depression). Participants will provide contact information via the online screening and the researcher will email participants to arrange an additional telephone screening interview. The latter is essential for developing rapport with participants and ensuring proper understanding of the commitment required for the study. Eligible participants will attend two sessions at UCL and perform the between-session tasks on consecutive days.  **Lab Sessions 1 and 2 (1 week apart)**  Sessions 1 and 2 are identical except that additional instructions for ‘at-home’ stimulation are provided at the end of Session 1.  Upon arrival, participants attach the relevant physiological probes (see Appendix V) which will continuously record heart rate. They then complete a series of *trait* questionnaires that assess mood (using the DASS-21 questionnaire), mindfulness, compassion, attachment security and childhood trauma (see Appendix V for full list). They also complete the baseline (i.e. Timepoint 1; T1) emotion recognition task (with simultaneous eye-tracking; see Appendix V) and pre-stimulation *state* measures of self-compassion (SCCS) and positive and negative affect (PANAS; Appendix V).  The researcher then familiarises the participant with the tVNS device and attaches the ear-clip to the participant’s ear (tragus or earlobe, depending on stimulation condition; See Appendix V for further detail) before starting the stimulation threshold-setting procedure. The threshold is set individually for each participant and corresponds to the current level at which s/he experiences a mild tingling sensation. The experimenter will explain the device settings to the participant. Participants then detach the ear-clip, switch the Parasym device off, before reattach the ear-clip and switching the device on and ensuring that the stimulator produces the same sensation as before. This is required to ensure that participants can use the device independently for the at-home stimulations.  Thirty minutes of constant tVNS is applied while the participant relaxes and listens to a standard set of (neutral) music pieces. After this, the emotion recognition/eye tracking task and state questionnaires are repeated (post-stimulation; T2), followed immediately by either the self-compassion or ‘draw-a-face’ imagery tasks (depending on the participant’s random assignment to imagery condition; see below) while stimulation continues. They then complete the final emotion recognition/eye tracking task, state questionnaires (post-imagery; T3) and questions on mental imagery vividness (Appendix V).  Any non-disposable equipment that involves direct contact with the participant’s skin will be disinfected with >70% ethanol solution and placed in a UV-C chamber for 11 minutes before use with the subsequent participant.  At the end of Session 1 (day 1), the experimenter provides instructions on the use of the tVNS device to the participant, and ensures that they understand the importance of retaining the stimulation settings and site of stimulation. The device is very easy to use and verbal instructions will be supplemented with brief written instructions to ensure compliance with the procedure. In fact, the main instructions are to *not modify* the settings from Session 1, and simply attach the ear-clip and switch the device on for the required period, as previously instructed. Participants return for Session 2 (day 8) one week later and repeat the above procedure.  **Between session stimulation and imagery**  From day 2 (the day after Session 1) until day 7 (the day before Session 2), participants use the tVNS device independently. Prior to stimulation they will log on to the Qualtrics study site and complete the baseline state questionnaires on affect and self-compassion. They then either attach the ear-clip to the tragus or the earlobe (depending on randomised condition used in Session 1) and initially stimulate for 30 min. The Qualtrics page will display a visual countdown indicating the required duration of stimulation. To maximise cooperation and minimise disruption to participants, no specific concurrent task is prescribed during the 30 min stimulation, other than suggesting that the participant sits and relaxes/ watches a nature documentary (i.e. activities that are minimally arousing).  After 30 min, participants proceed to the compassionate or control imagery audio instructions (which are the same as those they received on Session 1, according to randomisation) which are played via the Qualtrics site. Participants are free to select the volume level and mode of listening, i.e. via earphones (as long as these do not interfere with the ear-clip) or through their device’s loudspeaker. State affect and self-compassion measures are repeated after stimulation and after the mental imagery task.  **Note**: the Parasym device is a small battery operated ‘straight-to-consumer’ device, **designed to be used at home** independently of additional researcher/clinician instruction (see accompanying product information or details at [https://www.parasym.co/parasym-device-transcutaneous-vagus-nerve-stimulation.html](https://www.parasym.co/)). |
| --- | --- | --- |
|  | *Attach any questionnaires, psychological tests, etc.* *(a standardised questionnaire does not need to be attached, but please provide the name and details of the questionnaire together with a published reference to its prior usage).* |

| \| **B3** \| \| --- \| | **Where will the study take place (please provide name of institution/department)?** If the study is to be carried out overseas, what steps have been taken to secure research and ethical permission in the study country?  The work will be conducted at:   1. The Research Dept Clinical, Educational and Health Psychology (1-19 Torrington Place) and 2. The Psychology Department (26 Bedford Way)   Is the research compliant with Data Protection legislation in the country concerned or is it compliant with the General Data Protection Regulation 2018?  N/A. GDPR procures will be followed as required for studies conducted at UCL. |
| --- | --- | --- |

| \| **B5** \| \| --- \| | **How will the results be disseminated, including communication of results with research participants?**  The results will be disseminated in peer reviewed journals, conference presentations, and student dissertations. Participants interested in the findings will be informed of a rough timescale within which these reports might be published and invited to contact the researchers for the published report. |
| --- | --- | --- |

| \| **B6** \| \| --- \| | **Please outline any ethical issues that might arise from the proposed study and how they are be addressed.** *Please note that all research projects have some ethical considerations so do not leave this section blank.*  **Vagus nerve stimulation:** tVNS involves applying a small amount of electrical current (~5 mA) to the outer ear (tragus or earlobe). Participants only feel a mild tingling or pulsing sensation during stimulation. The stimulation ear-clips are easily removed if the participant feels any discomfort. Among the numerous published experimental lab studies in healthy people and in clinical trials, we are not aware of any reports of adverse reactions.  **Physiological measures/attachment of physiological probes:** Every effort has been made to limit skin-to-skin contact between participant and researcher. As such, the researcher will provide written and pictorial instructions on how participants can attach ECG electrodes to their torso themselves. In previous studies we have not found that data quality is adversely affected by participants attaching these devices themselves.  **Questionnaires**: A questionnaire used in this study – the childhood trauma questionnaire - enquires about distressing experiences of trauma (physical, sexual, emotional abuse) and neglect during childhood. The inclusion of this questionnaire is important because one possible moderator of the effects of tVNS (or self-soothing mental imagery) is the capacity to regulate autonomic activity, which can be adversely and enduringly affected by traumatic childhood experiences. We (and many other researchers) have used this questionnaire in numerous studies and have found it to be acceptable to participants. Nonetheless, responding to this questionnaire could potentially evoke unpleasant memories in some participants. As such, there is a specific statement in the participant information sheet stating that questions about difficult experiences from childhood will be asked during the study and that participants should not participate if they are likely to find such questions upsetting. The principal investigator is a clinical psychologist and registered psychotherapist and will be available for support and advice in the unlikely event of participants experiencing distress during the study.  The DASS-21 questionnaires inquire about anxiety and depression. Although we are not intending to recruit participants with significant levels of anxiety or depression, the DASS-21 may identify participants with likely depression and anxiety, which may require treatment. All participants, whether eligible or not, will therefore receive a mental health resource leaflet (attached; Appendix VI) at screening. In order to avoid stigmatising anyone, this leaflet will be given to all participants regardless of whether they show specific symptoms or not. Nonetheless, the researcher will un-intrusively draw participants’ attention to the leaflet at the end of screening if they score in the mild range or above for anxiety or depression. |
| --- | --- | --- |

| **SECTION C DETAILS OF PARTICIPANTS** |
| --- |

| \| **C1** \| \| --- \| | **Participants to be studied**   \| **C1a. Number of volunteers:** \| 140 \| \| --- \| --- \| \| Upper age limit: \| 35 \| \| Lower age limit: \| 18 \|   **C1b. Please justify the age range and sample size:**  Participants must be adults (hence the minimum age of 18). The upper limit will ensure that our sample is directly comparable to previous research in the field, including our recently completed study on tVNS (which used the same age range). In addition, ‘older’ women participants are more likely to experience hormonal changes (e.g. associated with menopause). Given that emotional processing can be affected by ovarian hormone levels, exclusion of older participants will reduce additional variability (noise) resulting from this. Clearly, ‘older’ men must also therefore be excluded to prevent imbalances in ages between sexes.  Sample Size: The sample size is based on pragmatic and statistical considerations. A sensitivity analysis suggests that n=140 will allow us to detect a between group effect size of d=0.48 (alpha=0.05; 1-beta=0.8; two tailed) for a critical comparison between active and sham tVNS groups on self-reported self-compassion (collapsed across imagery condition). This is a plausible effect size based on limited related research (e.g. d=0.6 in Petrocci et al, 2017, who examined the effects of anodal left temporal lobe transcranial Direct Current Stimulation on positive affect). We believe that tVNS’s effects on positive/affiliative mood states will be stronger than those found with tDCS (there is a stronger neurobiological rationale for tVNS), and as such, an effect size at least as large as observed by Petrocci et al (2017) can be expected in the current study. On this basis, an effect size d<0.5 is conservative, but seems appropriate given the uncertainties in the effects of tVNS on psychological functioning. Sensitivity analysis suggests that a lower sample size of n=120 is still sufficient to detect an effect size of 0.52. As such n=140 is aspirational and n=120 is acceptable. |
| --- | --- | --- | --- | --- | --- | --- | --- | --- |

| \| **C2** \| \| --- \| | **Accessing/Using Pre-Collected Data:**  **If you are using data or information held by a third party, please explain how you will obtain this. You should confirm that the information has been obtained in accordance with the General Data Protection Regulation 2018.**  NA |
| --- | --- | --- |

| \| **C3** \| \| --- \| | **Will the research include children or vulnerable adults such as individuals with**  **a learning disability or cognitive impairment or individuals in a dependent or unequal relationship?**  **Yes**  **No**    How will you ensure that participants in these groups are competent to give consent to take part in this study? *If you have relevant correspondence, please attach it.*  N/A |
| --- | --- | --- |

| \| **C4** \| \| --- \| | **Will payment or any other incentive, such as gift service or free services, be made to any research participant?**  **Yes**  **No**    If yes, please specify the level of payment to be made and/or the source of the funds/gift/free service to be used.  Upon completion participants will receive £70-80 (the final amount will be determined after the average time commitment has been determined upon piloting).  Please justify the payment/other incentive you intend to offer.  It is essential to properly compensate participants to ensure they are retained for the duration of the study and feel valued. This will increase the likelihood of compliance, which is especially critical for the between-session (at home) stimulation and imagery tasks. We anticipate that participants will commit 7-8 hr for the entire study. Specifically, they attend two lab sessions (~4 hr total) and complete 45 min of stimulation and imagery on days 2-6 (3.75 hr). The total compensation is based on £10/hr. All participants completing the study will receive the same amount based on the average amount of time taken to complete the study in piloting. |
| --- | --- | --- |

| \| **C5** \| \| --- \| | **Recruitment**  (i) Describe how potential participants will be identified:  Participants will respond to posts about the study. They will voluntarily respond by clicking on a web address which will direct them to study information or contact the researchers by phone to request information.  (ii) Describe how potential participants will be approached:  Participants will not be directly approached by researchers. They will learn about the study via posts on social media (e.g. Facebook); online experiment recruitment sites (e.g. Experimatch; Call for participants), UCL’s psychology participant pool; word-of-mouth; adverts on UCL notice boards; in-house participant mailing lists and flyers.  (iii) Describe how participants will be recruited:  Participants passing the online screening will provide their contact information to allow the telephone screening interview to be arranged. During the phone screening, researchers will provide more details about what is expected of participants and will answer any questions. The researcher will also confirm that participants meet the full eligibility criteria.  If the potential participant meets relevant criteria they will be invited to the first of two experimental sessions at UCL. At the start of Session 1 they will be given a printed copy of the information sheet and will be able to ask any outstanding questions about the study. They will then be asked for their signed consent. In consenting, all participants will be told that they can discontinue participation at any time without having to give a reason. |
| --- | --- | --- |

| \| **C6** \| \| --- \| | **Will the participants participate on a fully voluntary basis?**  **Yes**  **No**  **Will UCL students be involved as participants in the research project?**  **Yes**  **No**  *If yes, care must be taken to ensure that they are recruited in such a way that they do not feel any obligation  to a teacher or member of staff to participate.*  Participant will not be approached by the research team directly. The PI (the only member of staff on the research team who might be considered to be in a position of ‘power’) will have no role in recruiting participants.  **Please state how you will bring to the attention of the participants their right to withdraw from the study without penalty?**  This will be stated on both the information sheet and consent form and reiterated verbally at Session 1. |
| --- | --- | --- |

| \| **C7** \| \| --- \| | **CONSENT**  **Please describe the process you will use when seeking and obtaining consent.**  Participants will first be informed about details of the study via the online study site. After reading this information they will be asked if they wish to proceed to basic online eligibility screening. If eligible, based on basic criteria, a further telephone screening/information telephone call will be arranged with the researcher (the information sheet will be emailed to the participant prior to the telephone screening) to ensure full eligibility. Confirmation that the participant has read and understood the study information will be obtained by the researcher during this phone screening, and the researcher will ensure that participants are informed of and understand what is expected of them for the study. During this screening, the researcher will emphasise participants’ right to withdraw at any time without giving a reason.  If eligible, participants attend Session 1, at which they must confirm again that they have read and understood the information sheet prior to signing the study consent form.  *A copy of your participant information sheet(s) and consent form(s) must be attached to this application. For your convenience proformas are provided in Appendix I. These should be filled in and modified as necessary.*  In cases where it is not proposed to obtain the participants informed consent, please explain why below.  N/A |
| --- | --- | --- |

| \| **C8** \| \| --- \| | **Will any form of deception be used that raises ethical issues? If so, please explain.**  Although frank deception will not be used, it is necessary to conceal aspects of the study design and aims from participants because expectancy effects could invalidate the findings. In particular, participants are only informed that stimulation will be applied to the “outer ear” rather than the specific locations of stimulation (tragus or earlobe) to which they may be assigned. This is a *critical requirement* for the study, because if participants are told in advance that only one of two stimulation sites is expected to be ‘active’, they can easily obtain information about the active site and blinding would be removed. Interpretation of the findings would then be impossible as we would not be able to disentangle ‘real’ effects from expectancy-induced changes. As such participants will be ‘blind’ to the nature of the *active* stimulation.  On balance, given the need for incomplete disclosure of our aims and hypotheses, we believe it is necessary to include a statement on the use of deception in PIL. |
| --- | --- | --- |

| \| **C9** \| \| --- \| | **Will you provide a full debriefing at the end of the data collection phase?**  **Yes**  **No**  If ‘No’, please explain why below.  Full debriefing will occur at the end of session 2, including disclosure about the concealed aspects noted above. Participants will be requested not to discuss these debriefing details with others. |
| --- | --- | --- |

**ISKS AND BENEFITS tothe researcher and the researched**

| **SECTION D: APPROPRIATE SAFEGUARDS, DATA STORAGE AND SECURITY** |
| --- |

**SECTIO**

| \| **D1** \| \| --- \| | **Will the research involve the collection and/or use of personal data?**  **Yes**  **No**  ***Personal data*** *is data which relates to a living individual who can be identified from that data OR from the data and other information that is either currently held, or will be held by the data controller (the researcher).*  *This includes:*   - *any expression of opinion about the individual and any intentions of the data controller or any other person toward the individual.* - *sensor, location or visual data which may reveal information that enables the identification of a face, address, etc (some postcodes cover only one property).* - *combinations of data which may reveal identifiable data, such as names, email/postal addresses, date of birth, ethnicity, descriptions of health diagnosis or conditions, computer IP address (if relating to a device with a single user).*   **If yes, is the research collecting or using special category data as defined by the GDPR 2018**, for example data:   - - which reveals racial or ethnic origin, political opinions, religious or philosophical beliefs, trade union membership ;   - data concerning health (the physical or mental health of a person, including the provision of health care services) ;   - data concerning sex life or sexual orientation ; or   - genetic or biometric data processed to uniquely identify a natural person. - **data which might be considered sensitive in some countries, cultures or contexts?**   **Note that if you intend to process ‘special category’ information you will need an ‘additional’ legal basis for processing that particular data and further safeguards will need to be put in place.**  **If yes, state whether explicit ethical informed consent will be sought for its use and what data management measures are in place to adequately manage and protect the data.**  Our intention to collect personal data is explicitly stated on the information sheet and we will seek consent for collecting this data in the consent form.  Data management: All data are collected and stored electronically at source (via Qualtrics or physiological devices). Paper-based consent forms will be scanned and stored digitally as soon as practical, at which point, paper copies of these forms will be shredded.  Eligible participants will be assigned a participant number and as such, the data will be pseudonymous until data collection is complete. At that point, names / contact details will be securely deleted and the data will be fully anonymised. If participants have indicated that they would like to be contacted in the future for related research (per the original consent form), only their name, email address and phone number will be retained. Any reference to participation in the current study (including participant number) will not be retained with contact details. This information will be stored in a password protected file on a secure online UCL server.  While in pseudonymous form, the data will be stored on password protected spreadsheet files on secure UCL server-based storage. Participant names/contact details will be stored separately on a *single* password protected spreadsheet. The latter will *not* be transferred via email and there should be no occasion for this file to be copied or stored on any portable device. Individual digital data files (e.g. from the psychophysiological tasks/devices) will be stored securely. |
| --- | --- | --- |
| \| **D2** \| \| --- \| | **During the Project (including the write up and dissemination period)**  **State what types of data will be generated from this project** (i.e. transcripts, videos, photos, audio tapes, field notes, etc).  Questionnaire responses, physiological data (heart rate from ECG), reaction (and dwell) times collected on an eye tracking device (SR Instruments). All of this data will be in numerical form and contain no personal/identifiable information.  **How will data be stored, including where and for how long?** This includes all hard copy and electronic data on laptops, share drives, usb/mobile devices.  While pseudonymised, all electronic data will be stored on password protected spreadsheet files. As noted above, consent forms will be scanned, shredded and stored electronically on an encrypted mobile hard drive.  **Who will have access to the data, including advisory groups and during transcription?**  Only researchers directly involved in the research will have access to pseudonymised data during data collection. Once the data is fully anonymised it will be hosted on an appropriate open access research-data storage facility (e.g. Open Science Framework). |

| \| **D3** \| \| --- \| | **Will personal data be processed or be sent outside of the European Economic Area (EEA)*?**  **If yes,** please confirm that there are adequate levels of protection in compliance with the General Data Protection Regulation 2018 and state what these arrangements are below.  No |
| --- | --- | --- |

| \| **D4** \| \| --- \| | **After the Project**  **What data will be stored and how will you keep it secure?**  After the project, the data will be anonymous and will be stored electronically. Confidentiality will be guaranteed by anonymisation.  **Where will the data be stored and who will have access?**  Anonymous data will be stored on UCL and personal devices, and longer-term, it will be deposited in a data-sharing repository.  **Will the data be securely deleted?**  **If yes,** please state when will this occur:  Experimental data will not be deleted. |
| --- | --- | --- |

| \| **D5** \| \| --- \| | **Will the data be archived for use by other researchers?  Yes  No**  If **Yes**, please describe provide further details including whether researchers outside the EEA will be given access.  Data will be archived most likely on the Open Science Framework website. All researchers (worldwide) will have access to the anonymous archived data. |
| --- | --- | --- |
